# Brucella bacteremia in a recipient of an allogeneic hematopoietic stem cell transplant: a case report

**DOI:** 10.1186/1757-1626-2-91

**Published:** 2009-01-27

**Authors:** Khalid A Al-Anazi, Saleh Abu Jafar, Asma M Al-Jasser, Hamad Al-Omar, Fahad I Al-Mohareb

**Affiliations:** 1 Section of Adult Hematology and Hematopoietic, Stem Cell Transplant, King Faisal Cancer Centre, King Faisal Specialist Hospital and Research Centre, P.O. Box: 3345, Riyadh 11211, Saudi Arabia; 2 Section of Microbiology, Department of Pathology, Armed Forces Hospital, Box: X- 966, P.O. Box: 7897, Riyadh 11159, Saudi Arabia

## Abstract

**Background:**

Brucellosis is an important cause of morbidity and mortality in patients living in areas that are endemic for the infection.

**Case Presentation:**

A 20 years old Saudi male was diagnosed to have severe aplastic anemia at King Faisal Specialist Hospital and Research Centre in Riyadh in April 2006. One hundred and twelve days following his successful allogeneic hematopoietic stem cell transplant, he presented with pyrexia in addition to neutropenia and mild thrombocytopenia. Brucella serology was strongly positive and blood cultures grew Brucella melitensis. The bacteremic episode of brucellosis was successfully treated with streptomycin, doxycyclin and ciprofloxacin at the outpatient clinic. To our knowledge, this is the first case of a naturally occurring Brucella infection complicated by Brucella bacteremia in a recipient of hematopoietic stem cell transplant.

**Conclusion:**

Brucellosis may cause systemic infections, complicated bacteremias and serious morbidity in immunocompromised patients living in countries that are endemic for the infection. It should be considered as a possible cause of fever and pancytopenia in hematopoietic stem cell transplant recipients living in these geographical locations. Nevertheless, the infection is curable provided the diagnosis is made early and an appropriate antimicrobial therapy is promptly initiated.

## Background

Aplastic anemia (AA) was first described by Paul Enrlich in 1888 as hematopoiesis failure manifested by panytopenia and a fatty or an empty bone marrow. It can be effectively treated by either hematopoietic stem cell transplant (HSCT) or immunosuppressive therapy [1]. Antithymocyte globulin and cyclosporine-A can restore hematopoiesis in approximately two thirds of patients, but the recovery of the blood counts is often incomplete leading to: recurrent pancytopenia that requires re-treatment and certain late complications such as myelodysplastic syndrome. HSCT is usually a curative modality of therapy but is best used for younger patients who have histocompatible sibling donors [1].

Brucellosis, the commonest zoonotic infection in the world, can affect healthy individuals as well as immunocompromised hosts living in endemic areas [2-4]. Brucellosis and Brucella bacteremia (BB) have a wide range of clinical manifestations and complications may develop in case of delay in the diagnosis or in the institution of appropriate antimicrobial treatment [2,4-8]. Despite the seriousness of this infection in some instances, almost all the clinical manifestations and the laboratory abnormalities are reversible provided the diagnosis is established early and the appropriate antibiotic therapy is rapidly administered [4,5,7,8].

## Case presentation

A 20 year old Saudi male was diagnosed to have severe acquired AA at King Faisal Specialist Hospital and Research Centre (KFSH&RC) in Riyadh on 9/4/2006. He presented with 2 week history of low grade pyrexia, mucosal bleeding and anemic symptoms and his physical examination revelaed: pallor, few ecchymotic areas over the limbs, no external lymphadenopathy, a clear chest, no palpable abdominal organomegaly, and normal cardiovascular and neurological systems. Complete blood count (CBC) showed: WBC: 1.5 × 109/L with neutrophils of 0.02, Hb: 72 g/L and PLT: 5 × 109/L. Blood film revealed severe neutropenia and thrombocytopenia but no blast cells and bone marrow biopsy showed a hypocellular marrow with no blasts and a normal cytogenetic analysis. Renal, hepatic and coagulation profiles were normal. Screens for fanconi anemia, paroxysmal nocturnal hemoglobinuria, hepatitis, brucellosis, syphilis, viruses and autoimmune disorders were all negative. The patient was given supportive measures and after identifying an HLA identical and a healthy sibling donor, prepartions for allogeneic HSCT were made. The transplant conditioning was composed of fludarabine and cyclophophamide. The patient received acyclovir, fluconazole and trimethoprim-sulphamethoxazole (TMP/SMZ) as infection prophylaxis and cyclosporine-A and methotrexate as graft versus host disease (GVHD) prophylaxis. On 15/5/2006, the patient was given his allograft without any complication. The patient developed: grade I mucositis treated with IV morphine infusion and few febrile neutropenic episodes treated empirically with vancomycin, cefepime, amphotericin-B and metronidazole. However, no acute GVHD, cytomegalovirus infection, venoocclusive disease of the liver or hemorrhagic cystitis were encountered. The patient engrafted his leucocytes on day +24 and his platelets on day +16 HSCT. After the recovery of his blood counts, the patient was discharged on zantac, cyclosporine-A and prophylactic antimicrobials.

On 4/9/2006, he presented with 2 week history of high grade pyrexia, rigors and sweating. He denied intake of raw milk. Physical examination did not reveal any abnormality. CBC showed: WBC: 2.37 × 109/L with neutrophils of 1.59, Hb: 152 g/L and PLT: 97 × 109/L. Serum creatinine was 130 μmol/l [normal range: 0–122 μmole/L], bilirubin 30 μmol/L [normal range: 0–22 μmole/L], ALT: 194 U/L [normal range: 0–50 U/L], AST: 176 U/L [normal range: 0–40 U/L]. Blood cultures grew Brucella melitensis sensitive to: TMP/SMZ, streptomycin, tetracyclin, ciprofloxacin and imipenem. Brucella serology was strongly positive with 2-mercaptoethanol titer of 1:1280 and agglutination antibody titer of 1:20480. Initially, the patient was given streptomycin 1 gram intramuscularly twice daily and ciprofloxacin 400 mg orally twice daily for two weeks. Few days later, the clinical manifestation of brucellosis disappeared then the blood counts started to recover so streptomycin was replaced by doxycyline 100 mg orally twice daily and the patient was continued on ciprofloxacin for 4 more weeks. Subsequently, the blood cultures for Brucella became negative, the Brucella titers decreased significantly, the blood indices as well as the renal and the hepatic profiles normalized. When the patient was last seen on 29/4/2008, he was asymptomatic and his physical examination did not reveal any abnormality. CBC showed: WBC: 6.13 × 109/L with neutrophilis of 2.82, Hb: 142 g/L and PLT: 186 × 109/L. The renal and the hepatic profiles were normal. No new medication was prescribed and the patient was given a new follow up appointment.

## Discussion

Brucellae are Gram-negative strictly aerobic, nonmotile and nonencapsulated coccobacilli that can be isolated from the genitourinary tracts of several domestic and wild animals including: cows, goats, sheep, pigs and dogs [2]. Brucella abortus (B. abortus), B. suis, B. canis and B. melitensis are human pathogens that can cause systemic infections which may affect any organ system in the body thus resulting in a wide spectrum of clinical manifestations [2,7,9,10]. The epidemiology of human brucellosis has dramatically changed over the past 10 years due to various sanitary, socioeconomic and political factors. New foci of infection have emerged in Asia and the Near East while several traditionally endemic areas in Europe and South America have achieved control of the disease. Unfortunately, the disease is still present in some parts of Europe and the USA [3]. Direct contact with infected animals in addition to ingestion of unpasteurized milk and dairy products are the main routes of transmission of infection [2,11]. Contaminated aerosols can also transmit infection to abattoir workers, veterineans and laboratory technicians [2]. However, blood transfusion and HSCT are possible but very unusual means of transmission of brucellosis. Donors and recipients of blood and HSCT, living in areas that are endemic for the infection, should be screened for brucellosis and if they develop clinical manifestations suggestive of the infection, appropriate screening including blood cultures and Brucella serology should be done and anti-Brucella treatment should be administered without delay [11,12].

Brucellosis can cause a variety of clinical manifestations that include: fever, rigors, sweating, anorexia, malaise, weight loss, backache, bony pains, arthritis and arthralgias and hepatosplenomegaly [2,4-10,13]. Up to 90% of patients infected with Brucella develop BB. The great majority of patients with BB have positive Brucella serology with agglutination antibody titres of ≥ 1:320 [2,9]. Complications of BB include: infective endocarditis, fatal endotoxic shock, severe microangiopathic hemolytic anemia, disseminated intravascular coagulation (DIC), pancytopenia, bleeding tendency, multiorgan failure and death [9,10]. However, clinical improvement and normalization of blood indices as well as coagulation profiles may follow early administration of appropriate antimicrobial therapy [9,10,13].

Brucellosis is a leading infectious cause of pyrexia of unknown origin (PUO) in some parts of the world [4]. Brucella infections have been reported in patients with acute leukemia or solid tumors. Due to its slow growth in blood cultures and due to the late appearance of the clinical manifestations of Brucella infection, failure of empirical antibiotic therapy may be encountered in patients with febrile neutropenia. Therefore, brucellosis should be considered as a possible cause of febrile neutropenia and pancytopenia in immunocompromised hosts living in endemic areas [4,13].

Brucellosis may lead to various changes in hematological abnormalities eg anemia, leucopenia, thrombocytopenia and panytopenia in addition to bleeding disorders [5-8]. Anemia is the most frequently encountered abnormality and may occasionally be hemolytic in nature. The leucocytic count is usually depressed with predominant affection of neutrophils and lymphocytes [5,6]. The possible mechanisms involved in Brucella-induced thrombocytopenia are: immune destruction of platelets, reactive hemophagocytosis and hypersplenism [8]. DIC may also be seen in patients with severe infection [10]. The vast majority of patients with pancytopenia-induced by Brucella infection have positive blood cultures for the organism and almost all of these patients have positive Brucella serology with agglutination titres of ≥ 1:320 [2,7,9]. The bone marrow changes seen in patients with brucellosis are very variable and may include: hypercellular or nomocellular marrows, hemophagocytosis, granulomatous lesions, changes consistent with hypersplenism, reduced iron stores, abundant megakaryocytes in case of Brucella-induced thrombocytopenia and rarely pure red cell aplasia [5-8]. However, all the changes in the hematological parameters encountered in patients with brucellosis are often transient and reversible provided an appropriate antimicrobial therapy is initiated promptly [5,7,8,13].

Demonstration of high or rising antibody titers to Brucella antigen can make a presumptive diagnosis of brucellosis, but the infection can only be confirmed by culture of the organism from blood, bone marrow or other tissues. Brucella bacilli survive the intracellular killing by phagocytes and polymorphonuclear leucocytes and localize in the reticuloendothelial system thus leading to suboptimal recovery rate of the organism from blood, bone marrow, liver tissue or lymph nodes [2]. The use of aerobic bottles of the automated continuous-monitoring blood culture system, eg., BACTEC 9000 and BACTEC 9240 makes possible the diagnosis in more than 95% of positive blood cultures within 7 days thus obviating the need to perform subcultures of the negative media [2,14]. The Brucella ELISA test is a reliable and sensitive test in the diagnosis of brucellosis. It is rapid, easy to perform and can be automated [15].

The following antimicrobials have been employed in the treatment of brucellosis: TMP/SMZ, rifampicin, doxycycline, gentamycin and streptomycin [4,9,10,12]. The in-vitro resistance to TMP/SMZ is approximately 29%, but the overall rate of clinical relapse is about 5%. In uncomplicated cases of brucellosis, treatment for 6 weeks is usually sufficient, but the duration of therapy may be extended up to 3 months in case of complications such as spinal involvement or infective endocarditis [9].

The patient presented developed BB after having a successful allograft for his AA whilst having normal blood counts and being off all the transplant-related immunosuppressive medications. After acquiring the BB, his blood counts decreased significantly and the hepatic profile became impaired but his Brucella antibody titers were strikingly elevated reflecting his ability to mount a rather brisk immunologic response to the infection despite his reduced immunity. However, he responded very well to the treatment given and his blood indices and liver function tests reverted to normal levels after the control of the Brucella sepsis.

## Conclusion

Brucellosis can affect immunocompromised hosts as well as healthy individuals. Even recipients of various forms of HSCT can acquire this infection even after the recovery of their blood counts and after stopping their immunosuppressive therapy. Brucellosis should always be included in the differential diagnosis of febrile neutropenia and pancytopenia in immunocompromised hosts living in geographic areas that are endemic for brucellosis. Specific investigations including blood cultures for Brucella and Brucella serology should be taken and appropriate antimicrobial therapy should be initiated promptly. Brucellosis is curable and is usually responsive to antimicrobial therapy as the organism is susceptible to several antimicrobials, despite having some degree of resistance to trimethoprim-sulphamethoxazole.

## Abbreviations

Aplastic anemia; hematopoietic stem cell transplant; Brucella bacteremia; graft versus host disease; trimethoprim – sulphamethoxazole; pyrexia of unknown origin.

## Competing interests

The authors declare that they have no competing interests.

## Authors' contributions

All authors participated in the management of the patient presented. All authors read and approved the final form of the manuscript.

## Consent

Written informed consent was obtained from the patient for publication of this case report and accompanying images. A copy of the written consent is available for review by the Editor-in-Chief of this journal.
